# Quinolin-3-amine

**DOI:** 10.1107/S1600536812042626

**Published:** 2012-10-20

**Authors:** Arun M. Islor, B. Chandrakantha, Prakash Shetty, Thomas Gerber, Eric Hosten, Richard Betz

**Affiliations:** a National Institute of Technology-Karnataka, Department of Chemistry, Organic Chemistry Laboratory, Surathkal, Mangalore 575 025, India; b Manipal Institute of Technology, Department of Chemistry, Manipal 576 104, India; c Nelson Mandela Metropolitan University, Summerstrand Campus, Department of Chemistry, University Way, Summerstrand, PO Box 77000, Port Elizabeth, 6031, South Africa

## Abstract

In the crystal structur of the achiral title compound, C_9_H_8_N_2_, N—H⋯N hydrogen bonds connect the mol­ecules into zigzag chains in [100]. Weak inter­molecular N–H⋯π inter­actions further consolidate the crystal packing.

## Related literature

For novel applications of quinolin-3-amine and its derivatives, see: Rohmer *et al.* (2010 ▶); Kaneshiro *et al.* (2011 ▶). For the crystal structure of a rhodium coordination compound featuring the title compound as a ligand, see: Garralda *et al.* (1999 ▶). For graph-set analysis of hydrogen bonds, see: Etter *et al.* (1990 ▶); Bernstein *et al.* (1995 ▶).
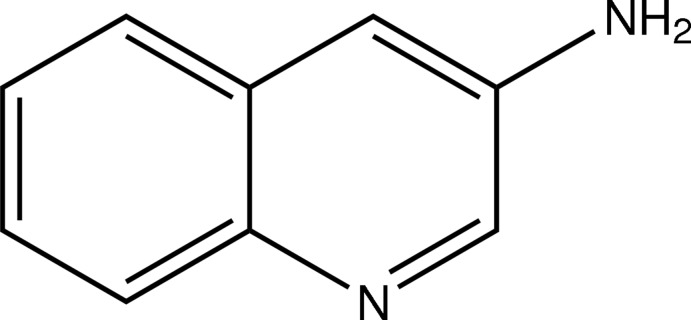


## Experimental

### 

#### Crystal data

C_9_H_8_N_2_
*M* *_r_* = 144.17
Orthorhombic, 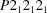

*a* = 7.6223 (3) Å
*b* = 7.6289 (3) Å
*c* = 12.6967 (4) Å
*V* = 738.31 (5) Å^3^
*Z* = 4
Mo *K*α radiation
μ = 0.08 mm^−1^
*T* = 200 K
0.55 × 0.52 × 0.15 mm

#### Data collection

Bruker APEXII CCD diffractometer
Absorption correction: multi-scan (*SADABS*; Bruker, 2008 ▶) *T*_min_ = 0.950, *T*_max_ = 0.988
6898 measured reflections
1077 independent reflections
1015 reflections with *I* > 2σ(*I*)
*R*_int_ = 0.013

#### Refinement

*R*[*F* ^2^ > 2σ(*F* ^2^)] = 0.032
*wR*(*F* ^2^) = 0.091
*S* = 1.03
1077 reflections
108 parameters
H atoms treated by a mixture of independent and constrained refinement
Δρ_max_ = 0.23 e Å^−3^
Δρ_min_ = −0.20 e Å^−3^

### 

Data collection: *APEX2* (Bruker, 2010 ▶); cell refinement: *SAINT* (Bruker, 2010 ▶); data reduction: *SAINT*; program(s) used to solve structure: *SHELXS97* (Sheldrick, 2008 ▶); program(s) used to refine structure: *SHELXL97* (Sheldrick, 2008 ▶); molecular graphics: *ORTEPIII* (Farrugia, 1997 ▶) and *Mercury* (Macrae *et al.*, 2008 ▶); software used to prepare material for publication: *SHELXL97* and *PLATON* (Spek, 2009 ▶).

## Supplementary Material

Crystal structure: contains datablock(s) I, global. DOI: 10.1107/S1600536812042626/cv5347sup1.cif

Supplementary material file. DOI: 10.1107/S1600536812042626/cv5347Isup2.cdx

Structure factors: contains datablock(s) I. DOI: 10.1107/S1600536812042626/cv5347Isup3.hkl

Supplementary material file. DOI: 10.1107/S1600536812042626/cv5347Isup4.cml

Additional supplementary materials:  crystallographic information; 3D view; checkCIF report

## Figures and Tables

**Table 1:** Hydrogen-bond geometry (Å, °) *Cg* is the centroid of the C1/C5–C9 ring.

*D*—H⋯*A*	*D*—H	H⋯*A*	*D*⋯*A*	*D*—H⋯*A*
N2—H2*B*⋯N1^i^	0.90 (2)	2.22 (2)	3.0761 (17)	158.2 (18)
N2—H2*A*⋯*Cg* ^ii^	0.85 (2)	2.60 (2)	3.3101 (15)	142.3 (19)

Symmetry codes: (i) 
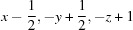
; (ii) 
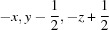
.

**Fig. 1.**
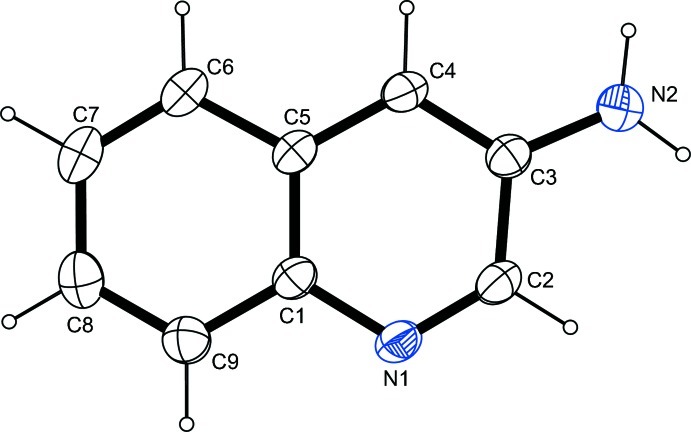
The molecular structure of the title compound, with atom labels and anisotropic displacement ellipsoids (drawn at the 50% probability level).

**Fig. 2.**
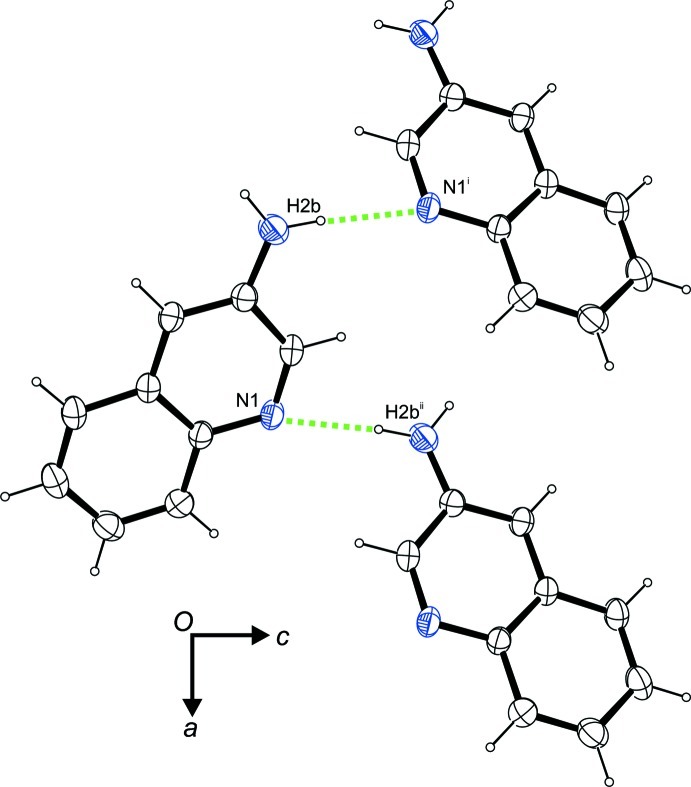
A portion of the crystal packing viewed along [010]. Dashed lines indicate N–H···N hydrogen bonds. Symmetry codes: (i) x - 1/2, -y + 1/2, -z + 1; (ii) x + 1/2, -y + 1/2, -z + 1.

## supplementary crystallographic information

### Comment

3-Aminoquinoline and its derivatives have found applications in matrix-assisted laser desorption ionization (MALDI) mass-spectrometry of oligosaccharides (Rohmer *et al.*, 2010) and glycans (Kaneshiro *et al.*, 2011). Herewith we present the crystal structure of 3-aminoquinoline, (I).

In (I) (Fig. 1), the molecule bears an amino group in *meta* position to the intracyclic nitrogen atom. Intracyclic angles in the six-membered ring containing the nitrogen atom cover a range of 117.42 (12)–125.27 (11) ° with the smallest angle found on the carbon atom bearing the amino group and the biggest angle present on the hydrogen-bearing carbon atom in *ortho* position to the intracyclic nitrogen atom. The molecule is essentially planar (r.m.s. deviation of of all fitted non-hydrogen atoms = 0.0091 Å). The least-squares planes defined by the non-hydrogen atoms of the heterocycle on the one hand and the atoms of the amino group on the other hand intersect at an angle of 11.97(2.58) °.

In the crystal, N–H···N hydrogen bonds (Table 1) are observed between the amino group and the intracyclic nitrogen atom that connect the molecules to zigzag chains along the crystallographic *a* axis (Fig. 2). In terms of graph-set analysis (Etter *et al.*, 1990; Bernstein *et al.*, 1995), the descriptor for these contacts is *C*^1^_1_(5) on the unary level. In addition, a N–H···π interaction (Table 1) involving the non-heterocyclic moiety of the quinoline core as acceptor contribute to the crystal packing stability.

### Experimental

To a solution of 3-nitroquinoline (1 g, 0.0057 mol) in methanol (20 ml) 10% palladium on carbon (0.10 g) was added. The batch was hydrogenated at a pressure of 10 bar for 12 h. Subsequently, the reaction mixture was filtered and concentrated under reduced pressure to afford the title compound as a pale yellow solid. The solid was dissolved in absolute ethanol and allowed to stand and evaporate at room temperature overnight. The crystalline solid that developed was filtered and dried under high vacuum (yield: 0.8 g, 97.5%).

### Refinement

C-bound H atoms were placed in calculated positions (C—H 0.95 Å) and were included in the refinement in the riding model approximation, with *U*_iso_(H) set to 1.2*U*_eq_(C). Both amino H atoms were located on a difference Fourier map and refined freely. In the absence of strong anomalous scatterers, 737 Friedel pairs were merged before the final refinement.

### Crystal data

**Table:**

C_9_H_8_N_2_	*D*_x_ = 1.297 Mg m^−^^3^
*M**_r_* = 144.17	Melting point = 366–368 K
Orthorhombic, *P*2_1_2_1_2_1_	Mo *K*α radiation, λ = 0.71073 Å
Hall symbol: P 2ac 2ab	Cell parameters from 5242 reflections
*a* = 7.6223 (3) Å	θ = 2.7–28.3°
*b* = 7.6289 (3) Å	µ = 0.08 mm^−^^1^
*c* = 12.6967 (4) Å	*T* = 200 K
*V* = 738.31 (5) Å^3^	Block, colourless
*Z* = 4	0.55 × 0.52 × 0.15 mm
*F*(000) = 304	

### Data collection

**Table:**

Bruker APEXII CCD diffractometer	1077 independent reflections
Radiation source: fine-focus sealed tube	1015 reflections with *I* > 2σ(*I*)
Graphite monochromator	*R*_int_ = 0.013
φ and ω scans	θ_max_ = 28.3°, θ_min_ = 3.1°
Absorption correction: multi-scan (*SADABS*; Bruker, 2008)	*h* = −6→10
*T*_min_ = 0.950, *T*_max_ = 0.988	*k* = −9→10
6898 measured reflections	*l* = −16→16

### Refinement

**Table:**

Refinement on *F*^2^	Primary atom site location: structure-invariant direct methods
Least-squares matrix: full	Secondary atom site location: difference Fourier map
*R*[*F*^2^ > 2σ(*F*^2^)] = 0.032	Hydrogen site location: inferred from neighbouring sites
*wR*(*F*^2^) = 0.091	H atoms treated by a mixture of independent and constrained refinement
*S* = 1.03	*w* = 1/[σ^2^(*F*_o_^2^) + (0.0591*P*)^2^ + 0.1011*P*] where *P* = (*F*_o_^2^ + 2*F*_c_^2^)/3
1077 reflections	(Δ/σ)_max_ < 0.001
108 parameters	Δρ_max_ = 0.23 e Å^−^^3^
0 restraints	Δρ_min_ = −0.20 e Å^−^^3^

### Special details

**Table:**

Refinement. Due to the absence of a strong anomalous scatterer, the Flack parameter is meaningless. Thus, Friedel opposites (737 pairs) have been merged and the item was removed from the CIF.

### Fractional atomic coordinates and isotropic or equivalent isotropic displacement parameters (Å^2^)

**Table:**

	*x*	*y*	*z*	*U*_iso_*/*U*_eq_	
N1	0.25487 (15)	0.26319 (16)	0.38612 (8)	0.0280 (3)	
N2	−0.18546 (17)	0.08305 (19)	0.39280 (11)	0.0359 (3)	
H2A	−0.258 (3)	0.023 (3)	0.3568 (15)	0.056 (6)*	
H2B	−0.198 (2)	0.098 (3)	0.4628 (17)	0.043 (5)*	
C1	0.30327 (17)	0.23241 (17)	0.28353 (9)	0.0249 (3)	
C2	0.09884 (17)	0.21121 (18)	0.41581 (9)	0.0277 (3)	
H2	0.0663	0.2323	0.4869	0.033*	
C3	−0.02593 (17)	0.12565 (16)	0.35016 (9)	0.0253 (3)	
C4	0.02155 (18)	0.09412 (17)	0.24707 (10)	0.0265 (3)	
H4	−0.0576	0.0376	0.2003	0.032*	
C5	0.18933 (16)	0.14693 (17)	0.21189 (9)	0.0244 (3)	
C6	0.24840 (19)	0.11883 (19)	0.10713 (10)	0.0299 (3)	
H6	0.1743	0.0609	0.0580	0.036*	
C7	0.4113 (2)	0.17449 (19)	0.07635 (10)	0.0344 (3)	
H7	0.4488	0.1553	0.0059	0.041*	
C8	0.52397 (19)	0.2599 (2)	0.14793 (11)	0.0353 (3)	
H8	0.6366	0.2982	0.1256	0.042*	
C9	0.47097 (17)	0.28794 (19)	0.25005 (11)	0.0313 (3)	
H9	0.5474	0.3448	0.2982	0.038*	

### Atomic displacement parameters (Å^2^)

**Table:**

	*U*^11^	*U*^22^	*U*^33^	*U*^12^	*U*^13^	*U*^23^
N1	0.0323 (5)	0.0323 (6)	0.0193 (5)	−0.0026 (5)	−0.0030 (4)	−0.0003 (4)
N2	0.0318 (6)	0.0443 (7)	0.0316 (6)	−0.0081 (5)	0.0052 (5)	−0.0086 (6)
C1	0.0277 (6)	0.0263 (6)	0.0207 (5)	0.0017 (5)	−0.0018 (5)	0.0009 (5)
C2	0.0340 (6)	0.0299 (6)	0.0191 (5)	−0.0004 (5)	−0.0012 (5)	−0.0007 (5)
C3	0.0279 (6)	0.0238 (5)	0.0243 (6)	0.0009 (5)	−0.0004 (5)	−0.0006 (5)
C4	0.0306 (6)	0.0264 (6)	0.0226 (5)	−0.0013 (5)	−0.0021 (5)	−0.0045 (5)
C5	0.0303 (6)	0.0228 (6)	0.0202 (5)	0.0027 (5)	−0.0006 (5)	−0.0003 (5)
C6	0.0382 (7)	0.0298 (6)	0.0218 (6)	0.0032 (5)	0.0015 (5)	−0.0037 (5)
C7	0.0430 (7)	0.0348 (7)	0.0254 (5)	0.0059 (6)	0.0084 (6)	−0.0008 (5)
C8	0.0320 (6)	0.0389 (7)	0.0350 (7)	0.0010 (6)	0.0070 (6)	0.0052 (6)
C9	0.0294 (7)	0.0347 (7)	0.0298 (6)	−0.0008 (5)	−0.0009 (5)	0.0008 (6)

### Geometric parameters (Å, º)

**Table:**

N1—C2	1.3091 (17)	C4—C5	1.4133 (18)
N1—C1	1.3740 (16)	C4—H4	0.9500
N2—C3	1.3701 (17)	C5—C6	1.4205 (17)
N2—H2A	0.85 (2)	C6—C7	1.369 (2)
N2—H2B	0.90 (2)	C6—H6	0.9500
C1—C9	1.4122 (18)	C7—C8	1.410 (2)
C1—C5	1.4167 (17)	C7—H7	0.9500
C2—C3	1.4231 (17)	C8—C9	1.375 (2)
C2—H2	0.9500	C8—H8	0.9500
C3—C4	1.3792 (17)	C9—H9	0.9500
			
C2—N1—C1	117.72 (11)	C4—C5—C1	118.88 (11)
C3—N2—H2A	119.4 (14)	C4—C5—C6	122.66 (12)
C3—N2—H2B	116.8 (12)	C1—C5—C6	118.45 (12)
H2A—N2—H2B	122.1 (18)	C7—C6—C5	120.52 (13)
N1—C1—C9	118.50 (12)	C7—C6—H6	119.7
N1—C1—C5	121.52 (12)	C5—C6—H6	119.7
C9—C1—C5	119.98 (11)	C6—C7—C8	120.78 (12)
N1—C2—C3	125.27 (11)	C6—C7—H7	119.6
N1—C2—H2	117.4	C8—C7—H7	119.6
C3—C2—H2	117.4	C9—C8—C7	120.04 (13)
N2—C3—C4	124.50 (12)	C9—C8—H8	120.0
N2—C3—C2	118.07 (11)	C7—C8—H8	120.0
C4—C3—C2	117.42 (12)	C8—C9—C1	120.22 (13)
C3—C4—C5	119.19 (12)	C8—C9—H9	119.9
C3—C4—H4	120.4	C1—C9—H9	119.9
C5—C4—H4	120.4		
			
C2—N1—C1—C9	179.86 (12)	C9—C1—C5—C4	−179.41 (11)
C2—N1—C1—C5	−0.20 (19)	N1—C1—C5—C6	−179.74 (12)
C1—N1—C2—C3	−0.3 (2)	C9—C1—C5—C6	0.20 (18)
N1—C2—C3—N2	−178.55 (13)	C4—C5—C6—C7	179.12 (12)
N1—C2—C3—C4	0.3 (2)	C1—C5—C6—C7	−0.5 (2)
N2—C3—C4—C5	178.95 (12)	C5—C6—C7—C8	0.3 (2)
C2—C3—C4—C5	0.15 (18)	C6—C7—C8—C9	0.1 (2)
C3—C4—C5—C1	−0.61 (18)	C7—C8—C9—C1	−0.4 (2)
C3—C4—C5—C6	179.80 (12)	N1—C1—C9—C8	−179.82 (13)
N1—C1—C5—C4	0.66 (18)	C5—C1—C9—C8	0.2 (2)

### Hydrogen-bond geometry (Å, º)

Cg is the centroid of the C1/C5–C9 ring.

**Table:**

*D*—H···*A*	*D*—H	H···*A*	*D*···*A*	*D*—H···*A*
N2—H2*B*···N1^i^	0.90 (2)	2.22 (2)	3.0761 (17)	158.2 (18)
N2—H2*A*···*Cg*^ii^	0.85 (2)	2.60 (2)	3.3101 (15)	142.3 (19)

Symmetry codes: (i) *x*−1/2, −*y*+1/2, −*z*+1; (ii) −*x*, *y*−1/2, −*z*+1/2.
